# Mapping the vulnerability of irrigation sand traps in a tropical volcanic basin, Indonesia

**DOI:** 10.1038/s41598-023-45036-z

**Published:** 2023-10-24

**Authors:** Ansita Gupitakingkin Pradipta, Ho Huu Loc, Edward Park, Akram Sripandam Prihanantya, Sigit Nurhady, Chandra Setyawan, S. Mohanasundaram, Salvatore G. P. Virdis, Sangam Shrestha

**Affiliations:** 1 Water Engineering and Management, Department of Civil and Infrastructure Engineering, School of Engineering and Technology, Asian Institute of Technology, Khlong Luang, 12121 Pathum Thani Thailand; 2 Department of Agricultural and Biosystems Engineering, Faculty of Agricultural Technology, Universitas Gadjah Mada, Sleman, 55281 Special Region of Yogyakarta Indonesia; 3 Water Systems and Global Change Group, Wageningen University and Research, Wageningen, The Netherlands; 4 National Institute of Education, Earth Observatory of Singapore and Asian School of the Environment, Nanyang Technological University, Singapore, 637616 Singapore; 5 Department of Geodetic Engineering, Faculty of Engineering, Universitas Gadjah Mada, Sleman, 55281 Special Region of Yogyakarta Indonesia; 6 Gama Tirtabumi Ltd., Yogyakarta, Indonesia; 7 Department of Information and Communication Technologies, School of Engineering and Technology, Asian Institute of Technology, Khlong Luang, 12121 Pathum Thani Thailand

**Keywords:** Civil engineering, Climate sciences, Environmental sciences, Natural hazards, Engineering

## Abstract

Sand traps in irrigation networks are typically used in mitigating canal sedimentation. In irrigation networks located in basins of high sediment yield due to the presence of volcanoes, it is essential to assess the vulnerability of sand traps. Using sediment yield at irrigation scheme inlets, sand trap vulnerability can be evaluated. This study aims to understand the vulnerability of irrigation sand traps throughout the Progo–Opak–Serang (POS) Volcanic River Basin, Indonesia, via mapping the sediment yield distributions in the basin. We employed the Revised Universal Soil Loss Equation to estimate soil loss, where the results show that the average soil loss in the POS River Basin is 179.69 tons/ha/year that falls under the category of moderate erosion potential, while the average sediment yield for the whole basin is 51.04 tons/ha/year. Parts of the basin with high yields of more than 180 tons/ha/year were mostly found along the volcanic mountains such as Sindoro, Sumbing, Merapi, Merbabu, and Telomoyo, and the Menoreh Hills. The model demonstrated relatively high performance with R^2^, NSE, RMSE, and MAE of 0.89, 0.82, 0.14, and 0.11, respectively. Within the POS Basin, Badran, Kalibawang, and Blawong are the three most vulnerable irrigation sand traps, with sediment yield values of 252.83, 178.92, and 63.49 tons/ha/year, respectively; they are all located in sub-watershed outlets. The vulnerability assessment conducted in this study can be used for the decision support system to prioritize irrigation sand traps towards a more effective irrigation system development.

## Introduction

**Figure 1:**
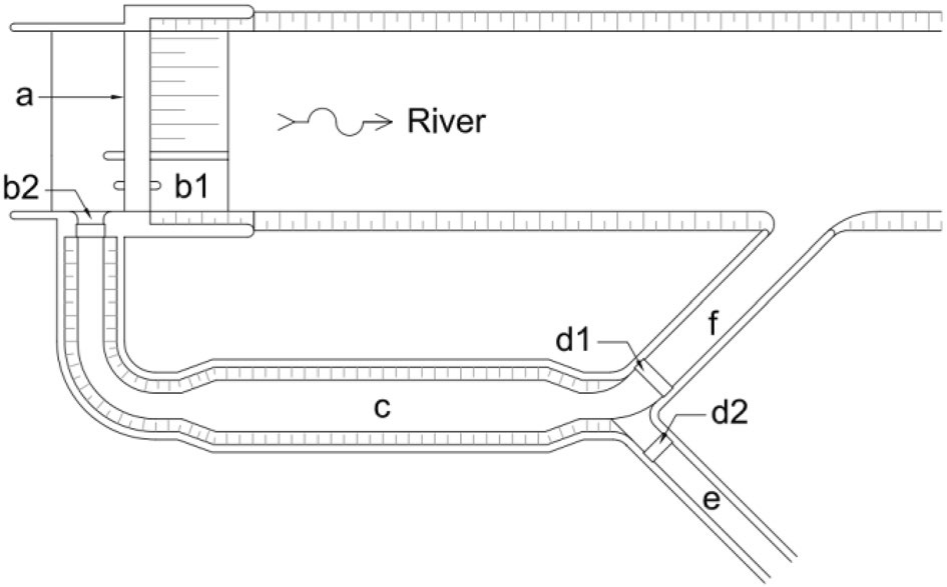
Typical layout of irrigation sand trap. It consists of (**a**) Weir, (**b1**) Flushing gate of the weir, (**b2**) Irrigation intake, (**c**) Sand trap, (**d1**) Flushing gate of the sand trap, (**d2**) Control gate to the primary irrigation channel, (e**)** Primary irrigation channel, (**f**) Flushing outlet.

A reliable irrigation system is essential for a country to implement a robust and significant national food system^1,2^. Irrigation is a technique to supply, manage, and release water to benefit the agricultural sector^3^. The benefits of irrigation are generally used to widely meet the water needs for agriculture, including livestock and fisheries^4,5^. Irrigation needs for rice plants still dominate the overall irrigation requirements in Indonesia, and it is delivered through a surface irrigation network^6^. The irrigation network is typically equipped with a sand trap-a device behind the irrigation intake-to deposit sediment carried from the river before it enters the primary irrigation channel^7,8^. Sand traps are commonly employed to enhance the efficacy of irrigation systems and have an important function for preventing or controlling canal sedimentation^9^. Immediately downstream of the irrigation intake is a sand trap fitted with a flushing gate^10,11^; its typical layout is shown in Fig. 1. The sediment deposited in the sand trap is cleaned on a frequent basis through manual or hydraulic techniques^9,10,12^. Sand trap is the initial barrier preventing sediment from the river before entering the irrigation network^8–10^. Therefore, insufficient sand trap performance might lead to sedimentation in the irrigation system. It will naturally reduce the irrigation conveyance efficiency^13^ and over the long term, this might represent a threat to agricultural productivity^14^. Around the world, irrigated areas contribute around 40% of agricultural production and 60% of crop yields^14–17^.

In irrigation networks located in the basins of high sediment yield due to the presence of volcanoes, it is essential to assess the vulnerability of sand traps^18^. The volcanic river basin refers to this particular river basin^19,20^. There are 133 river basins in Indonesia, each corresponding to one of the 34 River Basin Organizations. Among these, 127 are volcanic river basins^14^. Mount Merapi, one of the globe’s most active volcanoes, is situated in the Progo–Opak–Serang (POS) River Basin in Indonesia, hence the designation POS Volcanic River Basin^21^. In the past 200 years, Mount Merapi has erupted 41 times, with 15 of those eruptions being large. It typically erupts every three years, with major eruptions occurring every nine years^18^. The eruption material from volcanoes carried by debris flow affects river sedimentation^22^. Sedimentation causes siltation and damages sand traps and irrigation structures which draw water from the related river^23^.

Sediment yield at irrigation scheme inlets can be used to assess the vulnerability of irrigation sand traps. In general, sediment yield estimation is needed for soil and water conservation studies, including reservoir or irrigation channel sedimentation^24,25^. In Indonesia, there is limited study on sediment yield estimation in the volcanic river basin. A previous similar study had only examined a small-scale young volcanic catchment using a long-duration field measurement method and had been done 30 years ago^26^. It was discovered that the rainfed agricultural land contributed nearly half of the soil erosion, contributing to the sediment yield^26^. Updated research with a broader scope is needed that discusses the sediment yield pattern in the volcanic basin and its integration including the irrigation system. This can contribute to integrated water resource management. Another study recently conducted in 2019 modeled precipitation-runoff and sediment production at the Upper Opak Basin, that is portion of the POS River Basin. It was found that the highest sediment yield of the area from 2004 to 2013 was 147.8 tons/year. There is no further discussion regarding the comparison with other volcanic catchments or how this sediment yield relates to the irrigation system. In addition, there is no study on the vulnerability of irrigation networks based on sediment yield. Several existing studies discuss susceptible watersheds to sediment yield and sediment yield estimation to assess the area's vulnerability to soil erosion^27–32^.

There are several models used to predict soil erosion on a watershed scale, including empirical models, namely the universal soil loss equation (USLE) as well as its revised and modified versions (RUSLE and MUSLE)^33^; and a physical-based model, namely soil and water assessment tools (SWAT)^34^. USLE is a simple model with accessible and well-established parameters, so it is widely used in various parts of the world, while its disadvantage is that it cannot measure sedimentation^35–37^. RUSLE is a refinement of the USLE model, and this model will be better if combined with Geographic Information Systems (GIS)^25^. RUSLE is usually used for estimating annual erosion. The disadvantage of this model is that it cannot measure the amount of sediment produced^38^. MUSLE is a modified version of RUSLE that includes additional parameters that are not always accessible. The advantage of this model is that it can show the amount of sedimentation^39^. While SWAT is a complete model, it contains parameters of information about climate, soil properties, topography, plants, and land management contained in the watershed. It has been integrated with computers so that it is efficient to use, but one disadvantage is that the parameters used are quite large and must be filled when the model is operated, even though the desired results do not actually require these parameters^40^.

In this study, we map the sediment yield pattern that is able to estimate the vulnerability of irrigation sand traps throughout the POS Volcanic River Basin in Indonesia. We employed the revised universal soil loss equation (RUSLE) to forecast soil loss within the basin because of its ease of application and highly accurate projections predicting the quantity of erosion produced, making it one of the most extensively used models in research^38,41^. In conjunction with the geographic information system and remotely sensed data, the RUSLE model will generate more precise and reliable estimations^38,42^. The RUSLE model’s erosion prediction findings are utilized to estimate sediment yield patterns in the river basin^41,43–45^. The results can be used to determine the most vulnerable sand traps and prioritize them for sedimentation studies. It intends to investigate the current performance of sand traps as the principal sedimentation blocker in irrigation networks. Adequate sand trap performance can enhance the overall efficiency of the irrigation system. This study can serve as a resource for decision-makers in managing sedimentation in river basin-integrated irrigation networks.

## Methodology

### Study area

**Figure 2:**
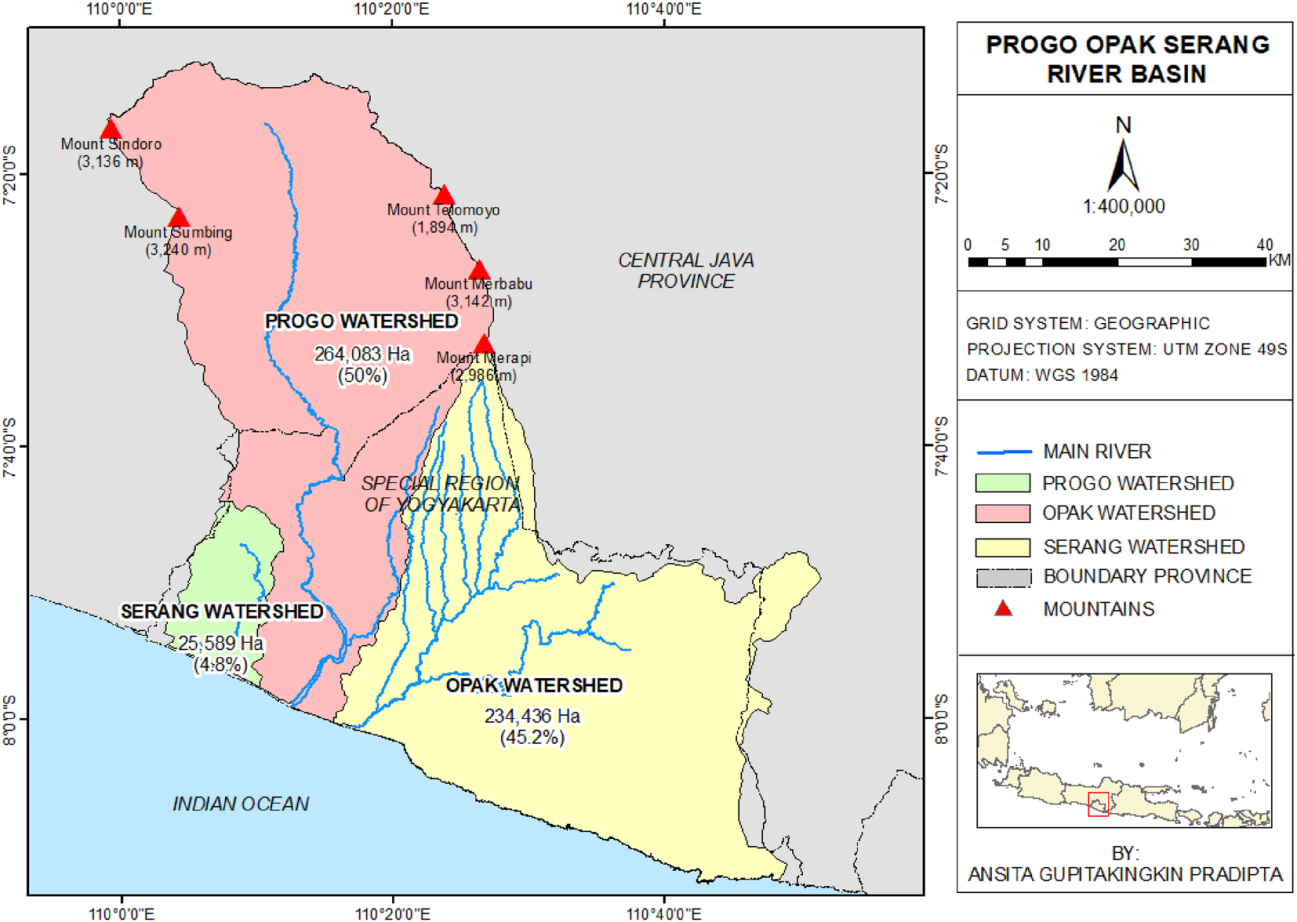
The study area: POS Volcanic River Basin, Indonesia. It is a basin comprised of three major watersheds: Progo, Opak, and Serang. This river basin contains Mount Merapi, one of the world's most active volcanoes, as well as other volcanoes, and is therefore known as the volcanic river basin^46^.

As illustrated by Fig. 2, this research was undertaken in the 5241-km^2^ POS Volcanic River Basin. POS is one of Indonesia’s interprovincial river basins, located in the Special Region of Yogyakarta (62.81%) and the Central Java Province (37.19%), and is governed by the Large River Basin Organization of Serayu-Opak (BBWS SO)^23^. BBWS SO manages two river basins, including the POS River Basin and the Serayu Bogowonto River Basin. The POS River Basin consists of three watersheds, namely Progo, Opak, and Serang, with 2640.83 km^2^, 2344.36 km^2^, and 255.89 km^2^. In general, the topographical condition of the POS River Basin consists of mountains and lowlands^23^. Mount Merapi, one of the globe's most active volcanoes, is situated in the POS River Basin. It has regularly erupted, and the activity has risen in the last 20 years. The eruptions have caused a lot of sedimentation, pyroclastic, and debris flows, which are dangerous to people and property in the area downstream^18^. Mount Merapi is located at 7° 32.5′ South and 110° 25.5′ East. It is 2986 m above sea level and 3079 m above the city of Yogyakarta^21^. Progo and Opak Watersheds particularly face problems related to the Mt. Merapi eruptions, aside from other related issues.

In addition to Mount Merapi, the POS River Basin is home to four more volcanoes: Mount Sindoro (3136 m MSL), Mount Sumbing (3240 m MSL), Mount Merbabu (3142 m MSL), and Mount Telomoyo (1894 m MSL). Nevertheless, these four volcanoes are classified as semi-active. Mount Sumbing last erupted violently in 1730, Mount Merbabu in 1797, and Mount Sindoro in 1910, while Mount Telomoyo has never erupted. This is unlike Merapi, which generally erupts every three to nine years, with the most recent explosive eruption in 2010. Because of its significance, Merapi is one of the world's sixteen volcanoes in the Decade Volcanoes project. The designation given by the International Association of Volcanology and Chemistry of the Earth's Interior to 16 volcanoes is deemed important for scientific study based on the history of large-scale and destructive eruptions and their location close to densely populated settlements. All these reasons make the POS River Basin fascinating to investigate. It can represent 126 other volcanic basins in Indonesia and other tropical regions in studies of sediment yields linked with irrigation systems.

### Methodological framework

**Figure 3:**
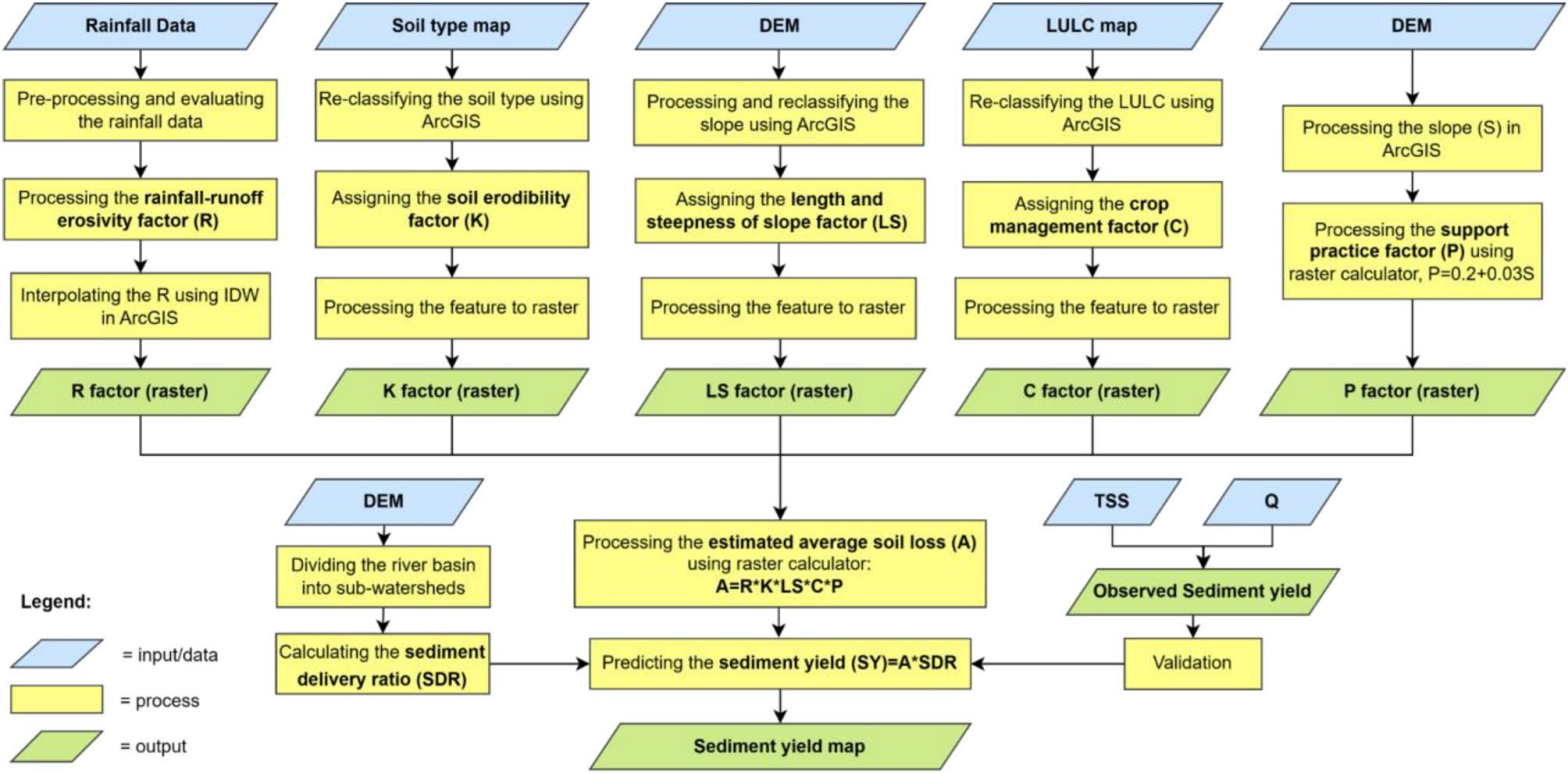
Overall methodological framework. The RUSLE is the preferred approach for predicting sediment yield throughout the POS River Basin.

This study uses the RUSLE model to predict average soil loss in the POS River Basin. It is used to estimate the distribution pattern of sediment yield in the river basin with the help of the sediment delivery ratio (SDR). The estimate of the SDR is derived from the delineation of the sub-watershed area within the basin derived employing the digital elevation model (DEM). The predicted findings of the sediment yield distribution obtained based on the model were then validated by using data pairs of total suspended solids and discharge at several measurement locations throughout the basin. Figure 3 illustrates the framework of this study.

### Data collection

The datasets used in the analysis were obtained from several institutions with the format, resolution, and time frame as shown in Table 1. Most data were collected through the Large River Basin Organization of Serayu-Opak (BBWS SO). This information includes tabular data on rainfall and daily discharge (gathered from automatic water level recorders, or AWLR) and vector maps of the POS River Basin's soil type and catchment area. We also collected data on total suspended solids at 10 locations within the basin from the Department of Environment and Forestry of the Special Region of Yogyakarta (DLHK DIY). Other raster maps are in the form of national DEM from the Geospatial Information Agency of Indonesia (BIG) and land cover from the European Space Agency (ESA).

**Table 1:** Data requirement.

No.	Data	Format	Temporal	Source
1.	Daily Rainfall Data			
	Progo: 54 stations, Opak: 25 stations, Serang: 4 stations	Tabular	1970–2020	BBWS SO
2.	Daily discharge data			
	Progo: 15 AWLR, Opak: 12 AWLR, Serang: 5 AWLR	Tabular	1990–2020	BBWS SO
3.	Total suspended solids (TSS) data at 10 Locations	Tabular	2013–2020	DLHK
4.	National DEM (DEMNAS) (Developed using various datasets: IFSAR (5 m), TerraSAR-X (5 m), and ALOS PALSAR (11.25 m), and by adding the stereo-plotting Masspoint data). Resolution: 0.27-arcsecond (± 8 m)	Raster (vertical datum EGM2008)	Released 2018	BIG
5.	Land cover, resolution: 10 m	Raster	2020	ESA
6.	Soil type	Vector	2005	BBWS SO
7.	Catchment area of the POS River Basin	Vector	–	BBWS SO

### Prediction of soil erosion

Due to data availability issues, RUSLE presented various ways of determining parameter values other than the original concept of USLE^47^. The RUSLE model is only capable of anticipating the rate of soil erosion due to sheet erosion and does not include trench erosion. The model cannot directly forecast sediment yield^34^. The RUSLE incorporates rainfall-runoff erosivity, soil erodibility, length and steepness of the slope, crop management, and supports practice factors. According to^48^, it is defined by Eq. 1.

1 $$A=R\times K\times LS\times C\times P$$

where *A* is average potential soil loss (tons/ha/year), *R is* rainfall-runoff erosivity factor (MJ·mm/ha/hour/year), *K* is soil erodibility factor (tons·hour/MJ/mm), *LS* is length and steepness of slope factor (dimensionless), *C* is crop management factor (dimensionless), and *P is* support practice factor (dimensionless).

*The rainfall-runoff erosivity (R)* component represents the soil's capacity being eroded and carried downstream as a result of precipitation intensity^48^. *R* is the total rainfall energy at a 30-min intensity, as computed by the equation *R* = *EI*_30_, in which *E* is the rainfall kinetic energy and *I*_30_ is the 30-min rainfall intensity^49^. Rainfall intensity is a significant element influencing *R* value; hence persistent precipitation data are necessary during *R* calculation. The *R* value is frequently derived from daily or monthly precipitation data^50^. An *R* value is computed in this study using the Lenvain equation using monthly rainfall in centimeters as an input (Eq. 2). It is derived on studies using precipitation data collected at various places on the Indonesian island of Java^51^.

2 $$R=2.21{{P}_{m}}^{1.36}$$

where *R* is rainfall-runoff erosivity factor (MJ·mm/ha/hour/year), *P*_*m*_ is monthly rainfall (cm)

For the Lenvain formula to estimate the value of *R*, the average monthly precipitation in centimeters is necessary. There are 83 rainfall stations within the POS River Basin, with 54 stations in the Progo watershed, 25 in the Opak watershed, and 4 in the Serang watershed. Each rainfall station has a different available data length. The Godean station, located in the Progo watershed, has the longest accessible data, spanning 46 years from 1978 to 2020. Before they can be used for *R* factor analysis, the entire rainfall data from all rain stations must be checked for consistency using a homogeneity test. Assessing the homogeneity of a time-series dataset of rainfall is critical for discovering data inconsistencies caused by non-climate-related variables, such as equipment failure, operator mistakes, and unexpected changes in the nearby region of the instrument^52^. In this study, the rescaled adjusted partial sums (RAPS) method was employed to deal with the homogeneity test.

*The soil erodibility (K)* factor expresses the soil's vulnerability to separation and transfer as a result of erosional energy^43^. *K* value can be measured directly using a Nomograph or a reference value. The Wischmeier and Smith method^48^, that incorporates soil nutrient content, structure, and permeability, is often used for direct measurements. For several soil types on Java Island, K values in this study were established using reference values, as stated in Table 2^53^.

**Table 2:** K rate (tons·hour/MJ/mm) for several soil types on Java Island, Indonesia.

Soil type	*K* rate	Soil type	*K* rate
Yellow–red latosol	0.560	Yellow podzolic	0.107
Grumusol	0.200	Yellow–red podzolic	0.320
Alluvial	0.470	Latosol	0.310
Regosol	0.400	Rensing and litosol complex	0.220

*The length and steepness of slope (LS)* factor indicates the correlation between slope length and steepness and soil erosion. The slope ratio was symbolized as *L*, which was identified as the slope length component approximated from the place where water flow first occurred above the soil surface to the site where precipitation first occurred^54^. *S* represents the slope steepness factor, which is given in slope angle degrees or percentage (%)^54^. In actuality, L and S variables are concurrently calculated as an LS component. Consequently, LS is referred to as the ratio of the amount of erosion on a surface area with a specific slope length and gradient compared to the identical land with a slope length of 22 m and a gradient of 9 percent^42^. Equation 3 can be employed to determine the LS's value.

3 $$LS={(x\left(0.0138+0.00965g+0.00138{g}^{2}\right))}^{0.5}$$

where *g* is slope steepness (%), *x* is slope length (meters)

Using Eq. 3, the slope steepness is used to get the *LS* component. Table 3 contains the *LS* categorization depends on the class of slope steepness^54^. The quantity of the *LS* component is established by examining the slope class map obtained from the DEM data analysis and then matched with the *LS* value table.

**Table 3:** Length and steepness of slope factor.

Slope (%)	LS index	Slope (%)	ls index
0–8	0.4	25–40	6.8
8–15	1.4	> 40	9.5
15–25	3.1		

*The crop management (C)* refers to the impact of vegetation cover on soil erosion^55,56^. As shown in Table 4, the C factor values range from 0 to 1, with 0 indicating that the area is completely protected from erosion^47^.

**Table 4:** C factor value for land use and land cover classes.

Land use and land cover	*C* factor value	Land use and land cover	*C* factor value
Built up land	0.004	Agriculture land	0.1
Dense forest	0.01	Wasted land	0.05
Open forest	0.014	Water body	0

*The support practice (P)* component represents the influence of soil erosion management techniques. *P* values vary from 0 to 1, with 1 signifying a region with no conservation practices, often known as undisturbed. We utilized the Wener Equation (Eq. 4) to estimate the *P* factor due to the inadequate observed data for river basin conservation efforts^57,58^. The DEM is used to generate the slope.

4 $$P=0.2+0.03S$$

where *P* is the support practice factor, *S* is the slope (%)

### Prediction of sediment yield

We estimated the sediment delivery ratio (SDR) that is necessary to forecast sediment yield at the catchment outlets^53,59^. This study calculated SDR based on the sub-watershed division within the POS River Basin. The procedure for delineating the sub-watershed using ArcGIS is using a DEM as an input and then creating fill sinks, flow direction, flow accumulation, pour points, and watersheds. After obtaining the sub-basin division, the SDR can be calculated using the formula adapted from the USDA SCS, as demonstrated in Eq. 5. We calculated the sediment yield (SY) by multiplying the RUSLE model results (spatial distribution of potential soil loss/A) by the SDR as demonstrated in Eq. 6^32,60^.

5 $$SDR=0.51\times {\left(0.386102\times {A}_{c}\right)}^{-0.11}$$

6 $$SY=A\times SDR$$

where *SDR is* sediment delivery ratio (dimensionless), *A*_*c*_ is catchment area (km^2^), *SY* is sediment yield (tons/ha/year), *A* is average potential soil loss (tons/ha/year).

### Model validation

The prediction of soil loss based on RUSLE needs to be validated using field measurement data. The data available is in the form of TSS at 10 locations in the POS River Basin. Most of these data were measured in tributaries of the Opak Watershed. In general, data is accessible for eight years, namely from 2013 to 2020. In each year, there are three data collection periods, namely in February, May, and September. Since the available TSS data is not continuous, developing a continuous data approach is necessary using the available daily discharge data. A rating curve depicting the connection between flow discharge (*Q*) and sediment discharge represents this method (*Qs*). For this reason, it is necessary to find a TSS measurement location where there is a discharge recording station using an AWLR. The POS River Basin has 32 AWLR stations: 15 in the Progo watershed, 12 in Opak, and 5 in Serang. Based on the investigation, eight TSS measurement sites are adjacent to the AWLR. The results of the TSS measurement (mg/L) are paired with the *Q* (m^3^/s) on the same day so that the *Q*-TSS data pair can be obtained. Then calculate *Q*_*s*_ with input *Q* and TSS as shown in Eq. 7.

After obtaining the *Q−Q*_*s*_ data pair, a rating curve can be made by the power regression. The rating curve is used to generate daily *Q*_*s*_ data along with the accessible daily *Q* data. Therefore, cumulative sediment volume (m^3^) can be obtained, converted into sediment load in tons/year, and finally converted into an average sediment yield in tons/ha/year. The results of the sediment yield measured at the location concerned are then compared with the value of the sediment yield at the same location derived from RUSLE modeling findings. The comparison is evaluated by performance indicators, namely coefficient of determination (R^2)^, Root Mean Square Error (RMSE), Mean Absolute Error (MAE), and Nash–Sutcliffe efficiency index (NSE).

7 $${Q}_{s}=Q\times TSS\times \frac{{10}^{3}}{{10}^{6}}\times \frac{1}{\rho }$$

where *Qs* is sediment discharge (m^3^/s), *Q* is river flow discharge (m^3^/s), *TSS* is total suspended solids (mg/L)*,* $\rho$ is the average density of sediment (kg/m^3^).

## Results and discussion

### Contributing Factors to soil erosion

**Figure 4:**
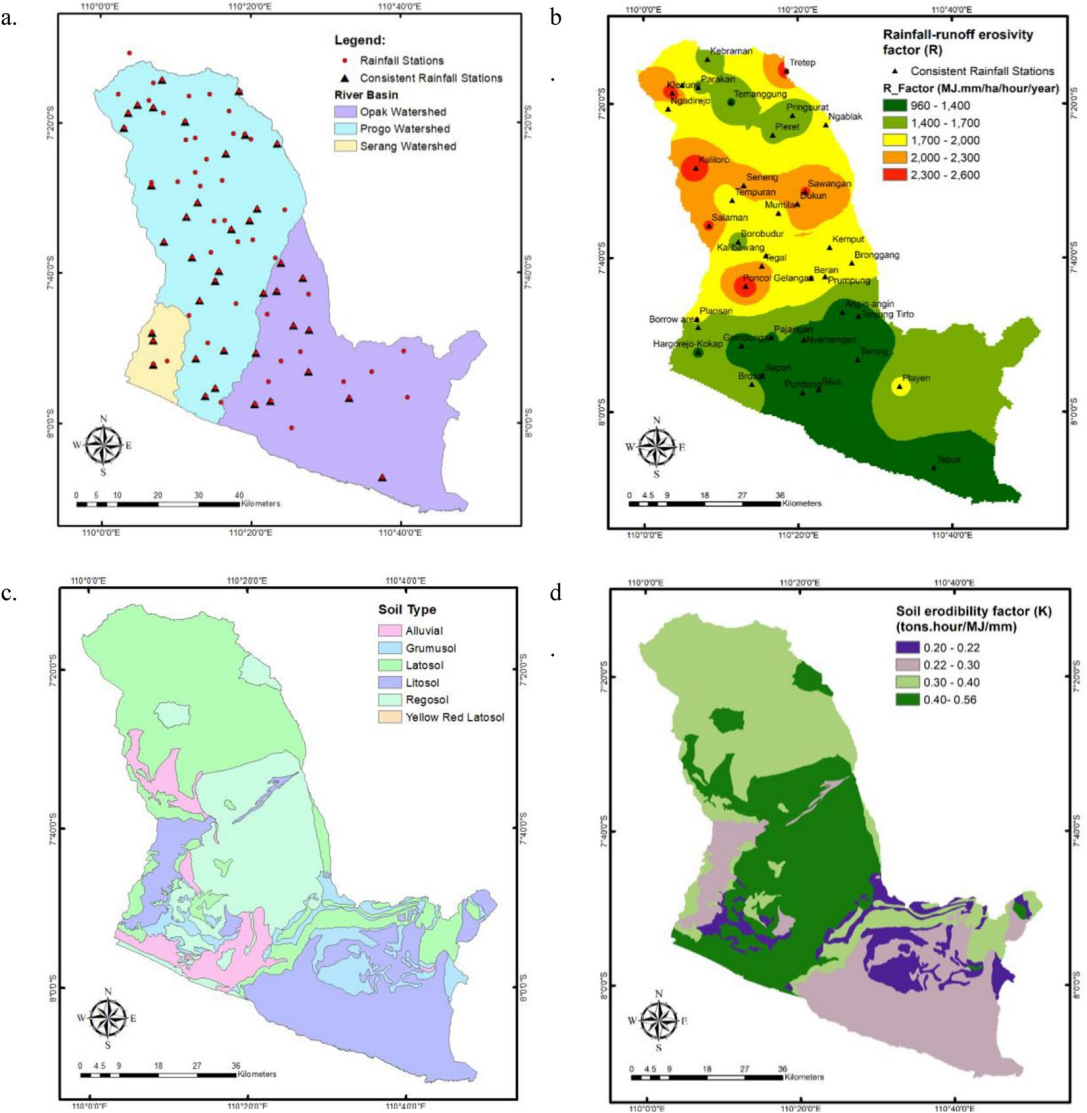

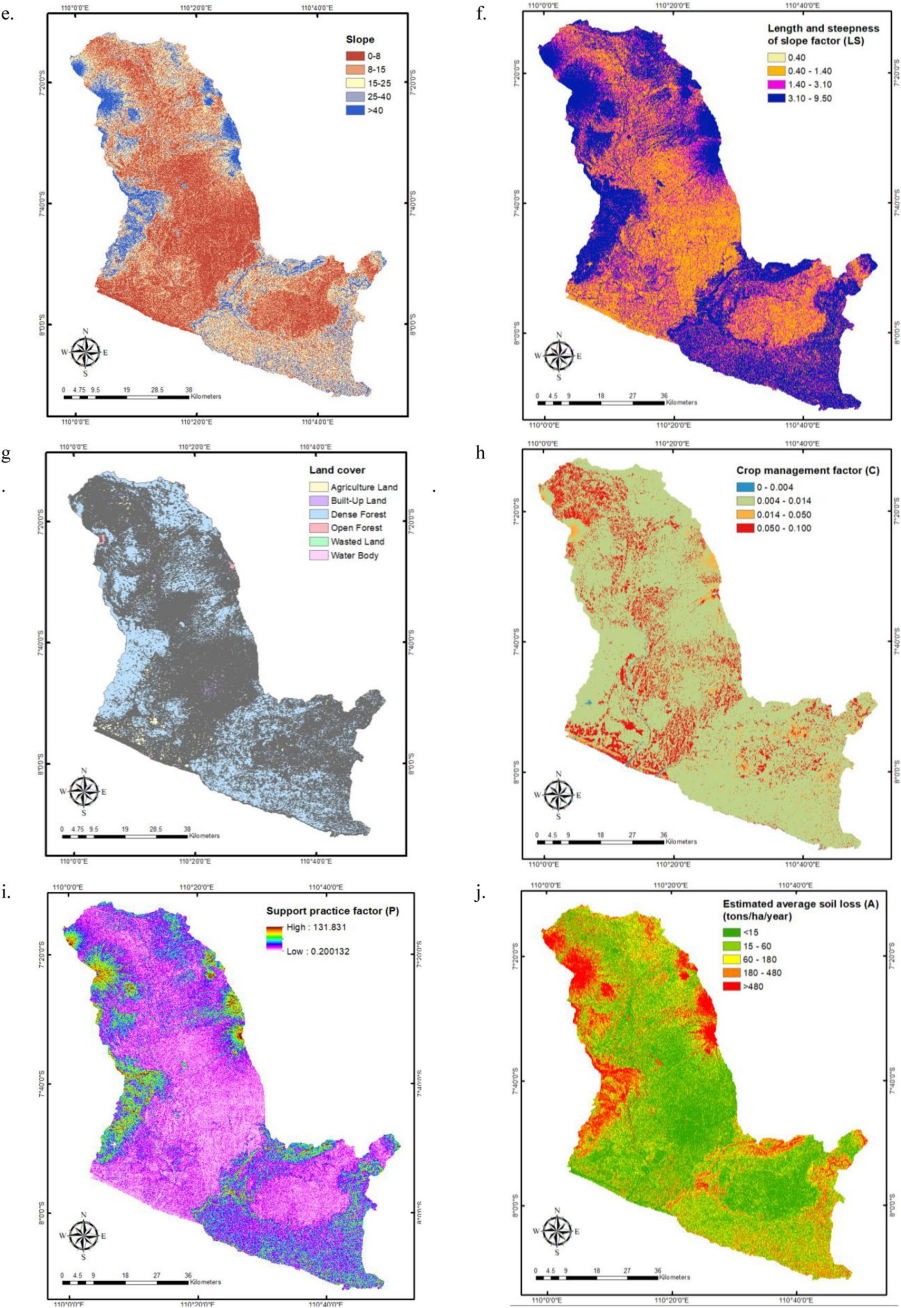
The spatial distribution of components and factors used in RUSLE within the POS River Basin. (**a**): The distribution of overall and consistent rainfall stations; no specific trend; (**b**): Rainfall-runoff erosivity factor (*R*) with an average value of 1656.66 MJ·mm/ha/hour/year; ©: Six distinct soil types, including alluvial, grumusol, latosol, litosol, regosol, and yellow–red latosol, with proportions of 7%, 8%, 36%, 25%, 24%, and 0.0001%; (**d**): *K* factor with high range of 0.40–0.56 tons·hour/MJ/mm is distributed in the middle and downstream parts of the Progo Watershed and upstream Opak; (**e**): five slope classes, including such as moderate (8–15%), moderately steep (16–25%), and steep (26–40%); (**f**): *LS* factor with an average value for the entire river basin is 2.72; (**g**): LULC map indicates that 73.61% of the POS River Basin is dense forest; (**h**): *C* factor with an average value is 0.022; (**i**): *P* factor spans from 0.2 to 131.8, with an average of 5.1; (**j**): The spatial pattern of the estimated average soil loss with an average of 179.69 tons/ha/year in the POS River Basin^46^.

The findings of the homogeneity test of rainfall data using the RAPS method indicate that 40 out of 83 rainfall stations have consistent data, including 25 stations in the Progo Watershed, 12 stations in the Opak Watershed, and 3 stations in the Serang Watershed (see Fig. 4a). There is no noticeable trend in the distribution of stations recording consistent precipitation. The inhomogeneity of the data is the result of missing or imperfectly recorded data in the field, which is the consequence of a variety of factors, including the negligence of recording officers, the damage to rainfall recording equipment caused by a lack of maintenance, and sudden changes in the condition of the equipment. In 2010, most rainfall data obtained at stations spread on the slopes of Mount Merapi or in the middle portion of the Opak Watershed and upstream of the Progo Watershed was not recorded. This is presumably related to the October 2010 eruption of Mount Merapi, but we lack sufficient information to be specific. In contrast to the Progo and Opak watersheds, where only half of the rainfall stations have consistent data, three out of four stations in the Serang have consistent data. This is since the rainfall station in the watershed were installed in the 2000s as opposed to the 1980s (such as Progo and Opak).

The consistent rainfall stations optimize the monthly precipitation and *R* factor. Figure 4b depicts R values interpolated with inverse distance weighted (IDW). It demonstrates that the average R factor value for the entire POS River Basin is 1656.66 MJ·mm/ha/hour/year. In the upstream and central regions of the Progo Basin, as well as in the upstream regions of the Opak watershed, high R values ranging from 1700 to 2600 MJ·mm/ha/hour/year can be found. This is related to the high monthly rainfall input in the region, which includes Mount Merapi, Mount Merbabu, Mount Sindoro, Mount Sumbing, Mount Telomoyo, and Menoreh Hills. Indonesia is positioned between two continents (Asia and Australia) as well as two oceans (Pacific and Indian). The monsoon circulation is caused by changes in physical attributes between the ocean and the continent^61^. As a monsoon region, Indonesia experiences considerable rainfall in the summer and autumn seasons. In monsoon regions, an orographic effect can enhance the amount of precipitation on the windward slope^61^. The windward slope is the side of a mountain, island, or other tall things that the wind hits. This causes some regions of the POS River Basin to experience intense rainfall, as it covers hilly and mountainous terrain. Moreover, maximum rainfall intensity data indicate that the west and southwest of Mount Merapi experience more precipitation than the southeast^62^. The south-eastern area of Mount Merapi is shown by the green space on the lower right in Fig. 4b.

The *K* value is strongly connected to soil physical parameters, including texture, structure, permeability, and organic content^50,63,64^. The erosion resistance of soils with a higher permeable level and a significant amount of organic matter is enhanced. Figure 4c depicts a map of the soil types within the POS River Basin with six distinct soil types, including alluvial, grumusol, latosol, litosol, regosol, and yellow–red latosol, with proportions of 7%, 8%, 36%, 25%, 24%, and 0.0001%, respectively. Latosols, litosols, and regosols dominate this river basin, with most latosol types covering the upstream area of the Progo Watershed, litosol covering the downstream portion of the Opak watershed and the upstream portion of the Serang watershed, and regosol covering the middle and upstream parts of both watersheds. A limited amount of yellow–red latosol was discovered in the Progo watershed's lower reaches. Based on the reference values of the *K* factor depicted in Table 2, we assigned the *K* quantities to every type of soil, and the spatial distribution of *K* values is demonstrated in Fig. 4d. High K concentrations of 0.40–0.56 tons·hour/MJ/mm are observed in the central and downstream portions of the Progo Basin, as well as upstream Opak. This refers to the regosol, alluvial, and yellow–red latosol soils with reference* K* values of 0.4, 0.47, and 0.56 tons·hour/MJ/mm, respectively. Regosols have weak structural cohesion and are common in eroding soils, especially in arid climate zones and mountainous regions^65^. Alluvial deposit, also known as alluvium, is loose clay, silt, sand, or gravel accumulated by moving water in a river bottom, in a floodplain, on an alluvial fan or seashore, or in other comparable environments^66^. This kind of soil is highly susceptible to erosion. Furthermore, there are seven classes of soil erodibility in Indonesia, namely very low (0.10 tons·hour/MJ/mm), low (0.10–0.15 tons·hour/MJ/mm), moderately low (0.15–0.20 tons·hour/MJ/mm), moderate (0.20–0.25 tons·hour/MJ/mm), moderately high (0.25–0.30 tons·hour/MJ/mm), high (0.30–0.35 tons·hour/MJ/mm), and very high (> 0.35 tons·hour/MJ/mm)^67^. The average values for soil erodibility in the Progo, Opak, and Serang watersheds are 0.34, 0.30, and 0.28 tons·hour/MJ/mm, respectively, while the average value in one river basin is 0.31 tons·hour/MJ/mm. Consequently, the POS river region falls into the category of highly erodible soils that have the potential to erode at a pace that is significantly larger than the ratio of soil loss considered tolerable.

To account for the *LS* component, we utilized the DEM-derived percentage slope. The slope of the terrain influences surface water runoff, recharge, and flow^68^. In general, it is classified into categories such as moderate (8–15%), moderately steep (16–25%), and steep (26–40%)^67^. Figure 4e depicts the slope class. The map shows that the Progo, Opak, and Serang Watersheds have average slopes of 16.95%, 18.74%, and 15.48%, respectively, while the whole river basin has a moderately steep slope of 16.38%. The Slope class was matched with Table 3's *LS* reference values. Figure 4f shows *LS* factor spatial distribution; it shows that the average *LS* factor for the river basin is 2.72, while Progo, Opak, and Serang Watersheds have 2.79, 3.26, and 2.58, respectively. Land slope distribution has a considerable impact on *LS* factor values and is one of the most important factors controlling the erosion process. *LS* increases the velocity of surface runoff, resulting in the separation of soil particles and erosion^69^. Understanding the slope and LS value distribution facilitates soil erosion control approaches that use engineering techniques such as terracing to lessen slope length and gradient impacts^47^.

*C* factor attempts to measure the contribution of management techniques (tillage, cover crops, and plant residues) to agricultural land soil loss^70^. When establishing the *C* component in RUSLE, land use and land cover classification are considered. Figure 4g indicates that 73.61% of the POS River Basin is dense forest, followed by 13.10% agricultural land and 7.09% built-up land. We assigned *C* values based on these categories, and Fig. 4h depicts the spatial distribution of *C* component. It shows that the average *C* component throughout the POS River Basin is 0.022, whereas the average *C* factor for every watershed is 0.024, 0.022, and 0.019 for the Progo, Opak, and Serang Watersheds, respectively. The *C* factor values range from 0 to 1, with a value of 0 indicating total protection against erosion. According to the average value of component *C*, it may be inferred that the POS river basin has adequate erosion protection. This is in accordance with Fig. 4g that most of the land is densely forested. Numerous ecosystem services are offered by forest, notably protection against soil erosion. Forests mitigate soil erosion by providing a surface cover using tree crowns that can reduce the kinetic energy of rainfall by intercepting precipitation^71^. Using the predicted value of the *C* factor, we can determine an area's susceptibility to erosion due to inadequate land management. Consequently, the *C* component is perhaps the most important aspect in policymaking and land use considerations, since it reflects the circumstances which are most easily managed to reduce erosion^70^.

*P* factor is the last component contributing to soil erosion in RUSLE. It describes the rate of soil loss produced by a particular soil conservation or protection method to slope loss^57^. It is a type of conservation used to evaluate the soil's space or rate of soil erosion with preservation treatments such as terracing, crop pattern, and total soil erosion controlled in accordance with the slope under the same conditions^42^. Limited in situ data prompted us to use a modified version of the Wener equation to estimate the P factor with the input parameter being slope in percent (Eq. 4)^57,58^.The spatial pattern of *P* component is illustrated by Fig. 4i; the value spans from 0.2 to 131.8, with an average of 5.1. Typically, *P* scores range from 0 to 1, with 1 being a territory without conservation efforts. The high *P* values result from the slope value, which is utilized as an input in the computation, is more significant than 100% at many locations in the POS river basin. A slope greater than 100% is feasible because some POS locations are mountainous and hilly. The location has a high slope degree, with a massive rise over a relatively short run, resulting in an extraordinarily high slope percentage.

All soil erosion factors are generated in raster format for use with a raster calculator to predict the average soil loss, and Fig. 4j displays the spatial pattern of the outcomes. The average soil loss in the POS River Basin is 179.69 tons/hectare/year, with 255.31, 159.24, and 96.73 tons/ha/year for the Progo, Opak, and Serang watersheds, respectively. In accordance with the Indonesian Department of Forestry's guidelines, the soil loss values can be classified according to Table 5^47^. The POS River Basin is under the category of moderate erosion potential, whilst the Progo watershed falls under the category of heavy erosion potential. According to^72^, 38% of the POS river basin was categorized as critical land in 2010, with an erosion rate of 235 tons/hectare/year. Compared to these findings, the erosion rate obtained in this study is realistic, and the POS River Basin has exhibited a declining trend in erosion risk over the previous decade. It should be emphasized, however, that each model will produce values that are not identical since they employ various inputs, methodologies, and assumptions. We also examined additional data in the Progo and Serang watersheds, although there were no comparative investigations in the Opak watershed. The average erosion rate in the Progo Basin increased from 165 tons/hectare/year just in 1999 reached 184 tons/ha/year in 2011^73^, whereas the erosion hazard level in the Upper Progo Sub-watershed in 2016 was 178.432 tons/ha/year^74^. According to^75^, the erosion value in the Serang watershed was between 195.866 and 5288.718 tons/ha/year in 2004, and the allowed erosion value in the Serang watershed ranged between 9.6 and 30 tons/ha/year.

**Table 5:** Soil Erosion Dispersion in the POS River Basin.

Erosion class (tons/ha/year)	Category	Coverage area (%)	Average erosion (tons/ha/yr)
< 15	Very low	31	5.01
15–60	Low	29	33.41
60–180	Moderate	16	108.88
180–480	Heavy	14	307.12
> 480	Very Heavy	9	1128.89

RUSLE, with the five factors that influence it, namely *R*, *K*, *LS*, *C*, and *P*, is a method that has been proven and well-established. The five factors determine the average potential soil loss in tons/ha/year. However, the magnitude of the contribution of each factor is different for various study areas in the world. In this regard, we conducted a correlation analysis to check how significant the five RUSLE factors are in calculating average potential soil loss in the POS River Basin. The correlation coefficient of RUSLE factors with average potential soil loss is shown in Table 6. The five factors (*R*, *K*, *LS*, *C*, and *P*) positively correlate with average potential soil loss (*A*). The highest coefficient value is shown by the *P* factor, followed by *LS* (0.054 and 0.0521). This results from the study area being dominated by hilly and mountainous areas, with high slope values. Furthermore, the *LS* factor is also closely related to the *P* factor, which is the main component in *P* factor mapping. The strong influence of the *LS* factor is in line with findings from other research, which show that the *LS* factor is one of the most significant agents contributing to soil erosion, representing the combined effect of slope steepness and length^76–78^.

**Table 6:** Correlation coefficient of RUSLE factors used to calculate the soil erosion in the POS River Basin.

	*R*	*K*	*LS*	*C*	*P*	*A*
*R*	1					
*K*	0.303	1				
*LS*	0.078	− 0.238	1			
*C*	0.019	0.139	− 0.200	1		
*P*	0.089	− 0.228	0.943	− 0.201	1	
*A*	0.199	0.010	0.521	0.184	0.540	1

*R*: rainfall-runoff erosivity factor (MJ·mm/ha/hour/year), *K*: soil erodibility factor (tons·hour/MJ/mm), LS: length and steepness of slope factor (dimensionless), *C*: crop management factor (dimensionless), and *P*: support practice factor (dimensionless), and *A*: average potential soil loss (tons/ha/year).

### Sediment yield distribution and validation

**Figure 5:**
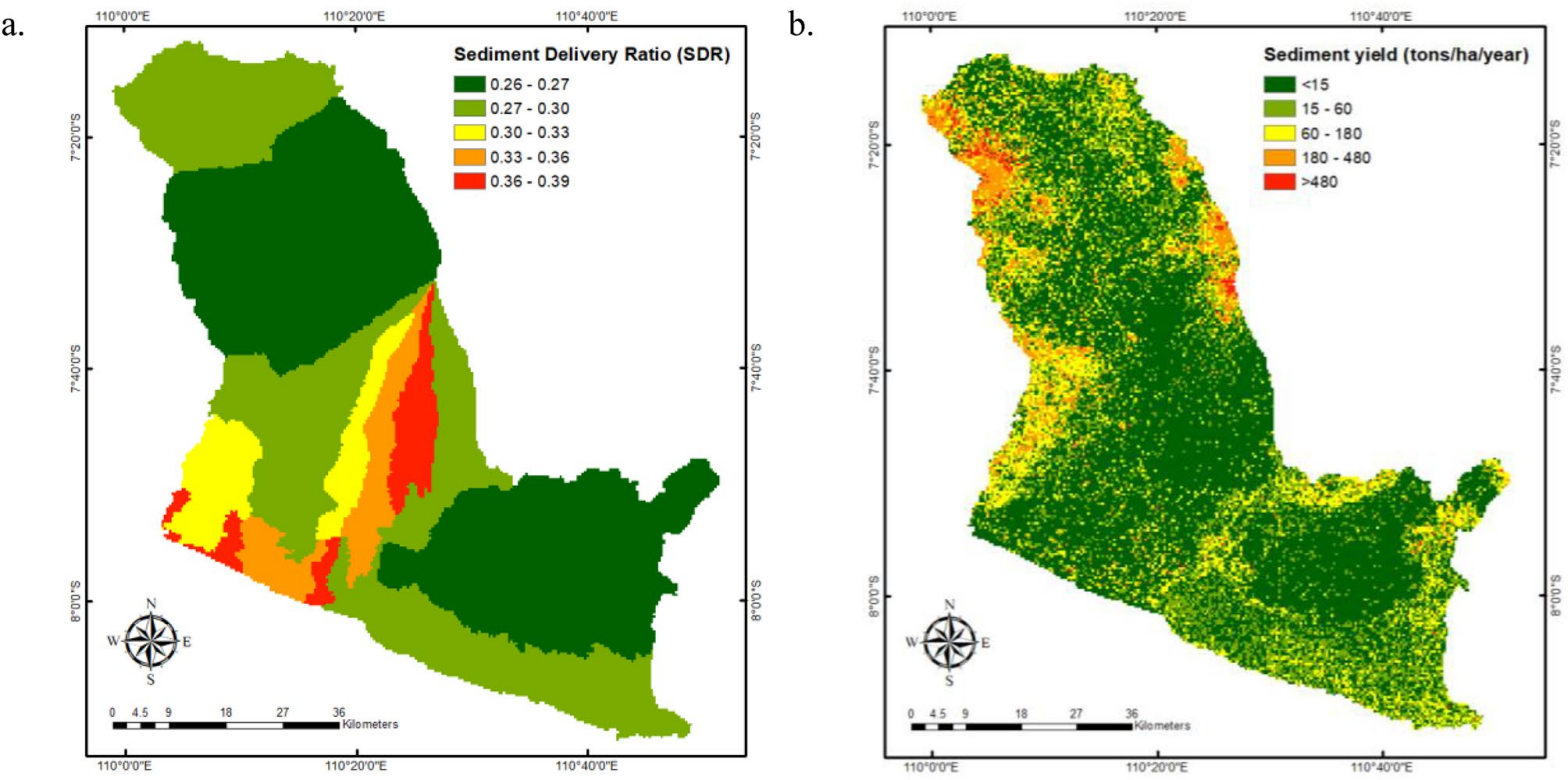
The spatial variability of SDR and sediment yield throughout the POS River Basin. (**a**): The distribution of SDR, with an average value of 0.28 within the river basin; (**b**): The distribution of sediment yield. The average sediment yield in the POS River Basin is 51.04 tons/ha/year, with values of 71.75, 50.23, and 27.87 tons/ha/year for the Progo, Opak, and Serang watersheds, respectively^46^.

The sediment delivery ratio (SDR) relates the quantity of sediments degraded and carried from a watershed’s gradient to the quantity that reaches the watershed’s outflow via streams and rivers^79^. SDR shows the relationship between the amount of erosion across the entire basin and the sediment yield at the watershed outlet^80^. The SDR value varies from 0 to 1, and a number close to one indicates that all soil moved by erosion enters the river. The assessment of annual sediment yield and sediment delivery has been a primary concern for watershed management authorities due to the impact of sedimentation on reservoir storage capacity and the yearly financial impact of disasters caused by sedimentation^79^. As depicted in Fig. 5a, the SDR was determined according to the division of the sub-watershed region in the POS River Basin. The average SDR within the POS River Basin is 0.28, and sub-watersheds originating in Mount Merapi, including the Bedog, Winongo, Gadjahwong, Code, Gawe, and Kali Kuning sub-watersheds, have the highest SDR values (0.33–0.39). Additionally, the downstream sub-watersheds of Progo, Opak, and Serang also have a high SDR. After obtaining the spatial variability of average soil loss (A) and SDR, the distribution of sediment yield in the river area can be estimated. The spatial pattern of sediment yield can be seen in Fig. 5a. The average sediment yield in the POS River Basin is 51.04 tons/hectare/year, with values of 71.75, 50.23, and 27.87 tons/ha/year for the Progo, Opak, and Serang watersheds, respectively, as shown in Fig. 5b. High sediment yields more than 180 tons/ha/year are mostly found in the Sindoro and Sumbing Mountains area, which are located upstream of the Progo watershed on the left; the Merapi, Merbabu, and Telomoyo Mountains, which are located in the middle of the Progo watershed and upstream of the Opak watershed on the right; and the Menoreh Hills, which are located in the middle of the Progo watershed and upstream of the Serang Basin on the left.

**Figure 6:**
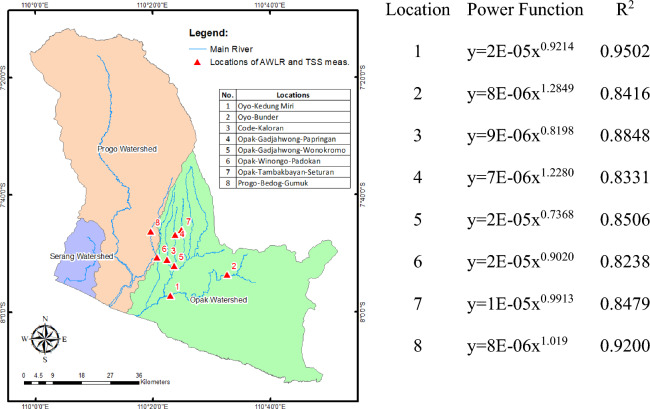
Locations of AWLR and TSS measurement throughout the POS River Basin. There are eight paired locations, seven of which are in the Opak Watershed. We also present the power function and R^2^ resulting from the rating curve creation at each location^46^.

To verify the accuracy of the RUSLE model technique-obtained framework, the sediment yield distribution was confirmed using observed data. Figure 6 represents eight total suspended solids (TSS) measurement locations that are subsequently converted to sediment discharges and linked with AWLR discharge data. Based on the power function, each location has a comparable rating curve pattern with a coefficient of determination greater than 0.80. Using the rating curve, we estimated the annual average sediment yield in tons/hectare/year. The sediment yield that was measured at the location of interest is then compared with the sediment yield that was predicted to be present at the same location based on the results of modeling with RUSLE. These results are presented in Table 7. The comparison is evaluated using R^2^, RMSE, MAE, and NSE and the results of the performance indicators are presented in Table 7. It is possible to conclude that the model has satisfactory accuracy because of the high values of R^2^ and NSE (both of which are greater than 0.8) and the low values of RMSE and MAE (0.89, 0.82, and 0.14, respectively). This study validates models using the TSS and Q methods, which are transformed into sediment yields using measurement data from most of the Opak Watersheds. We advise including measurement locations uniformly dispersed among the three watersheds. This will be highly beneficial for future sedimentation studies in the POS River Basin, which will assist in developing integrated water resource management. This must also be implemented in other watersheds in Indonesia, mainly volcanic basins, because, according to our observations, it is still challenging to gather sediment measurement data at the watershed scale, as opposed to hydro climatological data. The only watersheds where sedimentation measurements have been taken thus far are those with reservoirs. Institutions responsible for river basins, watersheds, water resources, and the environment are required to participate in the planning for sediment measurement at the watershed scale.

**Table 7:** Comparing observed sediment yield to expected sediment yield.

No.	Location of TSS measurement	AWLR	Sediment yield (tons/ha/year)		performance indicator	Value
			Observed	Predicted		
1.	Dogongan bridge	Kedung Miri	0.135	0.222	R^2^	0.8919
2.	Bunder bridge	Bunder	0.310	0.353	NSE	0.8209
3.	Ngoto bridge	Kaloran	1.313	1.248	RMSE	0.1432
4.	UIN bridge	Papringan	0.334	0.295	MAE	0.1152
5.	Kanggotan bridge	Wonokromo	0.363	0.391		
6.	Dongkelan bridge	Padokan	0.454	0.634		
7.	Jayakarta bridge	Seturan	0.471	0.687		
8.	Gamping bridge	Gumuk	0.273	0.535		

### Vulnerability of irrigation sand traps based on sediment yield

The vulnerability of the sand trap was evaluated based on criteria for sediment yields at the inlet of the irrigation network, i.e., at a location in the river basin before entering the weir with irrigation intakes. The greater the sediment yield at the entrance of the irrigation network, the more susceptible the performance of the irrigation sand trap is to sedimentation within the irrigation network. The susceptibility of this sand trap can be employed as a consideration in the decision support system (DSS) for prioritizing irrigation sand traps that are incorporated into a river basin for further performance-related research. For example^7^, evaluated the performance of the sand trap in the Wonji Shoa Sugar Estate Irrigation Scheme (Ethiopia)^81^, simulated the sand trap in the Sapon irrigation network (Indonesia)^82^, studied how to improve the performance of the sand trap in large scale irrigation scheme (Thailand)^83^, modeled the efficiency of settling basin in small scale irrigation projects (Kenya)^9^, assessed sand traps in the Pendowo and Pijenan Irrigation Scheme (Indonesia), while^8^ modeled sand trap behavior in the Pengasih Irrigation Scheme (Indonesia). These studies conclude that a sand trap's performance can be measured by capacity to hydraulically accumulate and flush sediment throughout regular flushing operations and that this will allow the sand trap to keep working correctly due to sedimentation problems in the irrigation network. Information on the current condition and performance of irrigation sand traps can be used as a reference in the management of integrated irrigation networks in a river basin, particularly in river basins that supply numerous irrigation networks.

In the POS River Basin itself, thousands of irrigation schemes draw water from the river, and these irrigation schemes can be classified into three categories of authority. The responsibility division for the region of authority is based on the criteria and areas stipulated in the Minister of Public Works and Housing Regulation of the Republic of Indonesia Number 14/PRT/M/2015, namely the existence of irrigation networks in administrative areas and strata of the irrigation network area. The responsibility division for the region of the authority of the irrigation schemes is based on the existence of the irrigation network in the strata of the irrigation network area, which includes central, provincial, and regency government authority irrigation schemes. Irrigation schemes with more than 3000 hectares, irrigation schemes across provinces or countries, or national strategic irrigation schemes are under the authority of the Central Government. Furthermore, irrigation schemes that have an area of 1000–3000 hectares or irrigation schemes across regencies or cities are under the authority of the Provincial Government. And finally, irrigation schemes with fewer than 1000 hectares and located in one regency/city area are under the authority of the Regency/City Government^8^. Based on the Ministerial regulation mentioned earlier, in the POS River Basin, there are 5, 45, and 1,235, respectively, the number of central, provincial, and regency government irrigation schemes, with a total irrigated area of 16,208, 18,081, and 37,986 hectares, respectively.

**Figure 7:**
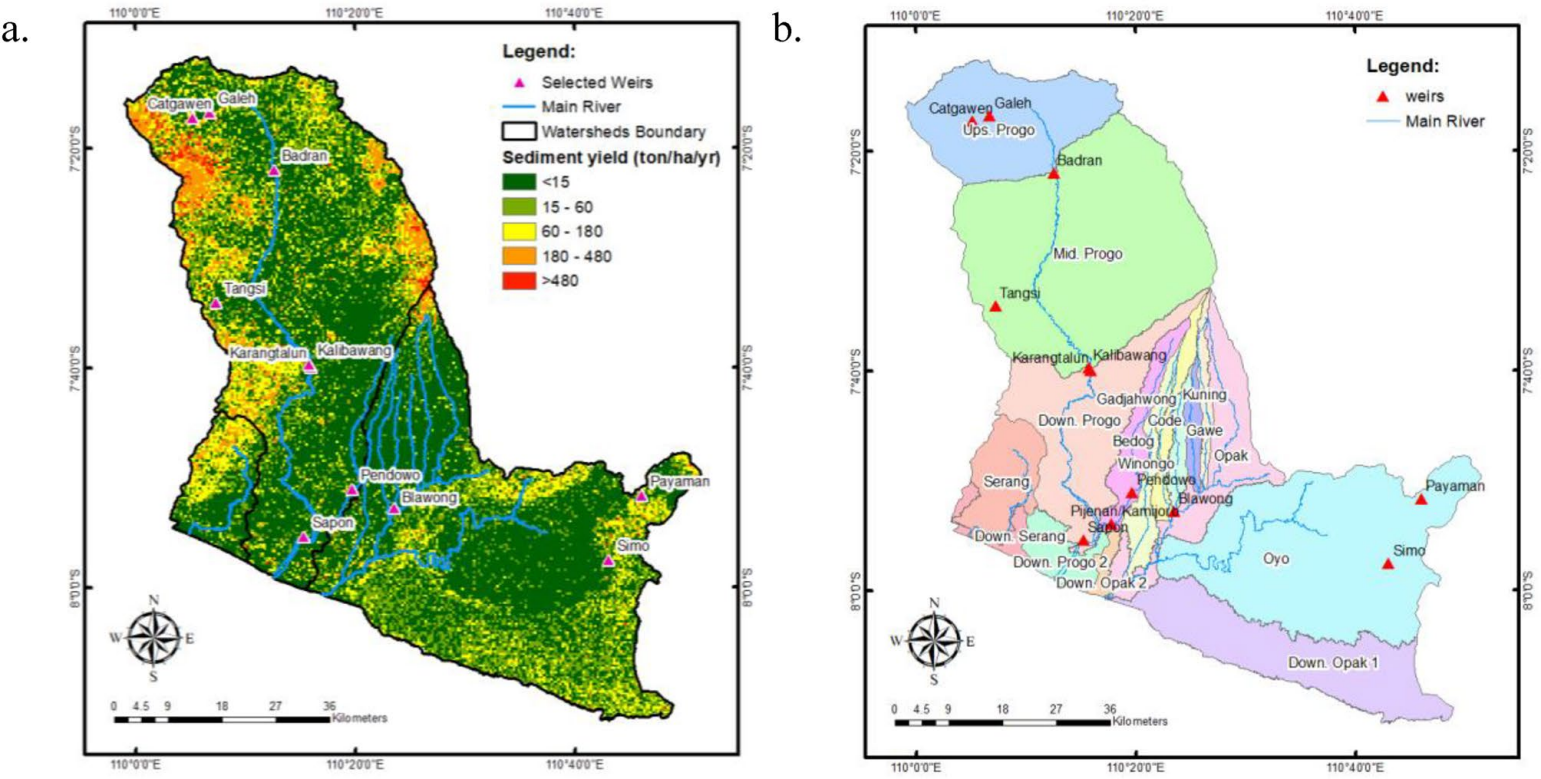
The spatial distribution of selected weirs on sediment yield map and sub-watersheds delineation. (**a**): On the sediment yield map, 12 weirs (the primary structure of irrigation networks) represent the location of irrigation networks. Based on the amount of sediment at the inlet, Badran, Kalibawang, and Blawong are the three most vulnerable sand traps in irrigation networks; (**b**): The location of weirs or irrigation networks inside sub-watersheds. Badran, Kalibawang, and Blawong are in the outlets of sub-watersheds^46^.

Based on the significance of their irrigated area, we only included the locations of irrigation networks under central and provincial government jurisdiction in this analysis. There are 3 irrigation schemes under the authority of the central government that fulfil the criterion of a service area greater than 3000 hectares; the remaining two are cross-provincial irrigation schemes with an insignificant service area of 149 and 115 ha. In addition, there are 9 irrigation schemes under the jurisdiction of the provincial government with a service area of between 1000 and 3000 hectares. At the same time, the remaining 36 are cross-regency irrigation schemes with a range of 1 to 541 hectares, which is negligible. On the POS River Basin sediment yield map, we displayed 12 selected irrigation schemes based on authority and service area, as demonstrated in Fig. 7a. They were represented by the weir's position, the irrigation network's main structure, or the inlet of sediment yield from the river system. Regarding Fig. 7a, the three most vulnerable sand traps of irrigation networks are Badran, Kalibawang, and Blawong, with sediment yield values of 252.82, 178.92, and 63.49 tons/ha/year at these locations, respectively. Badran and Kalibawang gain water from the Progo River, whilst Blawong receives water from the Opak river system. The sediment yield at the intake of nine other irrigation networks is insignificant, ranging from 0.47 to 40.38 tons/ha/year. Figure 7b illustrates the sub-watersheds within the POS River Basin; we can observe that Badran is located at the Upstream Progo outlet, Kalibawang at the Middle Progo outlet, and Blawong at the Winongo outlet, or the junction of the Winongo and Opak Rivers. This implies that the massive sediment yields at all three locations are acceptable. After the prioritization is based on this sediment yield estimate, a field investigation must be conducted to determine the actual condition of sedimentation at the irrigation network's intake. This assessment should include irrigation-related institutions, operators, and water user organizations.

A greater number of stakeholders may be engaged, and irrigation system management can be conducted more efficiently. The broader perspective provided by a river basin can capture dimensions that are normally not included in the regulation of irrigation systems, such as the factors for the scarcity of water, the quality of the water, legal problems involving the usage of water, and unequal water distribution and use. From this study, it can be inferred that river basins, including volcanoes, have a more significant potential for sedimentation than plain areas. Consequently, planning for integrated water resource management (IWRM) in a volcanic river basin necessitates a distinctive strategy. The IWRM components paradigm posits two governance layers: policy-making and management environment. These layers are positioned at the national, regional, and local levels of government. The initial module includes the political context within the scope of water resource management legislation in Indonesia. The second layer illustrates the interrelationships between stakeholders and their responsibilities in the IWRM aspects, which are structured as governance levels and management pillars: Water conservation, utilization, and risk management^19^. An integrated approach to water resources management in a river basin will increase both the productivity and sustainability of natural resource usage, contributing to the accomplishment of sustainable agriculture and food security in compliance with Sustainable Development Goals 2 and 6.

In addition, this study represents an important advance in mapping the vulnerability of irrigation sand traps based on sediment yield patterns, particularly in river basins influenced by volcanism. Before this study, no other research had been conducted that addressed this specific issue, even though there are numerous volcanic river basins around the globe, 127 of which are in Indonesia. This study demonstrates that the volcanic river basin has the characteristics of a high average sediment yield, with specific hilly and mountainous areas averaging more than 180 tons/ha/year. The factor that contributes the most to the high sediment yield is the high value of the slope’s length and steepness. This must be considered when formulating an IWRM strategy for volcanic river basins. This addresses the sedimentation problem in downstream water resource infrastructure, including the irrigation network. A network of irrigation systems with minimal sediment has high conveyance efficacy. This will positively affect agricultural productivity, particularly rice, Indonesia's staple diet. It will improve the welfare and social stability of the people in the study area whose primary livelihood is farming. This aligns with the first Sustainable Development Goal: to end all forms of poverty everywhere.

## Conclusion

The average soil loss in the Progo-Opak-Serang (POS) Volcanic River Basin is 179.69 tons/ha/year; it falls under the category of moderate erosion potential. The average sediment yield in the POS River Basin is 51.04 tons/ha/year. High sediment yields of more than 180 tons/hectare/year are mainly concentrated in the Sindoro, Sumbing, Merapi, Merbabu, and Telomoyo Mountains areas and the Menoreh Hills. The model has good accuracy, identified by R^2^, NSE, RMSE, and MAE values of 0.89, 0.82, 0.14, and 0.11, respectively. The significance of the sediment yield identified at the inlet of the irrigation network can be one of the criteria for assessing the vulnerability of irrigation sand traps within the POS River Basin. The greater the sediment yield at the entrance of the irrigation network, the more susceptible the performance of the irrigation sand trap is to sedimentation within the irrigation network. The susceptibility of this sand trap can be employed as a consideration in the decision support system (DSS) for prioritizing irrigation sand traps that are incorporated into a river basin for further performance-related research. Badran, Kalibawang, and Blawong are the three most vulnerable irrigation sand traps, with sediment yield values of 252.83, 178.92, and 63.49 tons/ha/year at these locations; they are all located in sub-watershed outlets. Furthermore, a field investigation must be conducted to determine the actual condition of sedimentation at the irrigation network's inlet. This assessment should include irrigation-related institutions, operators, and water user organizations. A more significant number of stakeholders may be engaged, and irrigation system management can be conducted more efficiently. Furthermore, planning for integrated water resource management (IWRM) in a volcanic basin necessitates a specific strategy because it is more susceptible to sedimentation than plain regions.

## Data Availability

Most of the data supporting this study's findings is available from the Large River Basin Organization of Serayu-Opak (BBWS SO). Restrictions apply to the availability of the data, which were used under license for this study. Data are available from the corresponding author upon reasonable request and with the permission of the BBWS SO. While some data are openly available on the Department of Environment and Forestry of the Special Region of Yogyakarta (DLHK DIY) website https://dlhk.jogjaprov.go.id/, the Geospatial Information Agency of Indonesia (BIG) website https://tanahair.indonesia.go.id/demnas/#/, and the European Space Agency (ESA) https://www.esa.int/.

## References

[1] Rockström J (2017). Sustainable intensification of agriculture for human prosperity and global sustainability. Ambio.

[2] Borsato E, Rosa L, Marinello F, Tarolli P, D’Odorico P (2020). Weak and strong sustainability of irrigation: A framework for irrigation practices under limited water availability. Front. Sustain. Food Syst..

[3] Batubara, R. D., Amelia, V. & Barbara, B. Economic value of irrigated water in Gunung mas regency of central Kalimantan. **11**(30) 1–5 (2021)

[4] Angelakis AN (2020). Irrigation of world agricultural lands: Evolution through the Millennia. Water (Switzerland).

[5] Boelee E, Laamrani H, van der Hoek W (2007). Multiple use of irrigation water for improved health in dry regions of Africa and South Asia. Irrig. Drain..

[6] Vermilion, D. L., Lengkong, S. R. & Atmanto, S. D. Time for Innovation in Indonesia’s Irrigation Sector. [Online]. Available: https://www.oecd.org/greengrowth/sustainable-agriculture/49175235.pdf. Accessed 5 Oct 2022.

[7] Paulos T, Yilma S, Ketema T (2006). Evaluation of the sand-trap structures of the Wonji-Shoa sugar estate irrigation scheme, Ethiopia. Irrig. Drain. Syst..

[8] Pradipta AG (2022). Mathematical modeling-based management of a sand trap throughout operational and maintenance periods ( case study: Pengasih irrigation network, Indonesia ). Water (Switzerland).

[9] Widaryanto LH (2018). Evaluation on flushing operation frequency of sand trap of Pendowo and Pijenan weirs. J. Civ. Eng. Forum.

[10] Pradipta, A. G., Maulana, A. F., Murtiningrum, Arif, S. S. & Tirtalistyani, R. Evaluation of the sand trap performance of the Pengasih weir during the operational period. In *IOP Conference Series Earth Environmental Science* **542**(1). 10.1088/1755-1315/542/1/012053 (2020).

[11] Richter W, Vereide K, Mauko G, Havrevoll OH, Schneider J, Zenz G (2021). Retrofitting of pressurized sand traps in hydropower plants. Water (Switzerland).

[12] Mustafa, M. R., Tariq, A. R., Rezaur, R. B. & Javed, M. Investigation on dynamics of sediment and water flow in a sand trap. In *International Conference on Mechanics, Fluids, Heat, Elasticity, and Electromagnetic Fields*, pp. 31–36. 10.46300/9105.2020.14.3 (2013)

[13] Bhatti MT, Ashraf M, Anwar AA (2021). Soil erosion and sediment load management strategies for sustainable irrigation in arid regions. Sustainability (Switzerland).

[14] Ahmad MD, Turral H, Nazeer A (2009). Diagnosing irrigation performance and water productivity through satellite remote sensing and secondary data in a large irrigation system of Pakistan. Agric. Water Manag..

[15] Ungureanu N, Vlăduț V, Voicu G (2020). Water scarcity and wastewater reuse in crop irrigation. Sustainability (Switzerland).

[16] Siebert S, Döll P (2010). Quantifying blue and green virtual water contents in global crop production as well as potential production losses without irrigation. J. Hydrol. (Amst).

[17] Leng G, Leung LR, Huang M (2017). Significant impacts of irrigation water sources and methods on modeling irrigation effects in the ACME Land Model. J. Adv. Model. Earth Syst..

[18] Jazaul, I. *Study on Integrated Sediment Management in an Active Volcanic Basin*. Dissertation (Kyoto University, 2010). [Online]. Available: 10.14989/doctor.k15649.

[19] Ariyanti V, Edelenbos J, Scholten P (2020). Implementing the integrated water resources management approach in a volcanic river basin: A case study of Opak Sub-Basin, Indonesia. Area Dev. Policy.

[20] Ariyanti V (2019). Governing a Volcanic River Basin: A Culture-Sensitive Inquiry into the Current Water Resources Management Practices of Opak Sub-Basin, Indonesia.

[21] Purwadi, H., Bayuadji, T. & Ariyanti, V. Addresing food, energy and water nexus in a volcanic area. In *2nd World Irrigation Forum*, pp. 1–8 (2016).

[22] Ikhsan J, Kurniati R, Harsanto P, Nursetiawan (2020). Analysis of sediment transport on the upstream code river, Indonesia. Civ. Eng. Archit..

[23] Sukardi, S., Warsito, B., Kisworo, H. & Sukiyoto, *River Management in Indonesia*. (Directorate General of Water Resources: Jakarta Pusat, 2013).

[24] Ijaz MA (2022). Prediction of sediment yield in a data-scarce river catchment at the sub-basin scale using gridded precipitation datasets. Water (Switzerland).

[25] Kandpal, K. C. & Joshi, J. Estimation of sediment yield of Randigad Catchment, Pauri District, (Uttarakhand), India, using remote sensing and GIS techniques. *Int. J. Adv. Inf. Sci. Technol. (IJAIST)* **7**(8) (2018).

[26] Rijsdijk, A. Evaluating sediment sources and delivery in a tropical volcanic watershed. In *Sediment Budgets I*, Foz do Iguacu 1–9 (IAHS Publication, 2005).

[27] Jain MK, Das D (2010). Estimation of sediment yield and areas of soil erosion and deposition for watershed prioritization using GIS and remote sensing. Water Resour. Manag..

[28] Kefay T, Abdisa T, Tumsa BC (2022). Prioritization of susceptible watershed to sediment yield and evaluation of best management practice: A case study of Awata River, Southern Ethiopia. Appl. Environ. Soil Sci..

[29] Browning, T. N. & Sawyer, D. E. Vulnerability to watershed erosion and coastal deposition in the tropics. 10.1038/s41598-020-79402-y (2021). 10.1038/s41598-020-79402-y PMC7806860 33441573

[30] Dibaba WT, Demissie TA, Miegel K (2021). Prioritization of sub-watersheds to sediment yield and evaluation of best management practices in highland Ethiopia, Finchaa Catchment. Land (Basel).

[31] Panda C, Das DM, Raul SK, Sahoo BC (2021). Sediment yield prediction and prioritization of sub-watersheds in the Upper Subarnarekha basin (India) using SWAT. Arab. J. Geosci..

[32] Vemu S, Pinnamaneni UB (2012). Int. Arch. Photogramm. Remote Sens. Spatial Inf. Sci..

[33] Noori H, Siadatmousavi SM, Mojaradi B (2016). Assessment of sediment yield using RS and GIS at two sub-basins of Dez Watershed, Iran. Int. Soil Water Conserv. Res..

[34] Susanti, Y., Syafrudin, S. & Helmi, M. Soil erosion modelling at watershed level in Indonesia: A review. In *E3S Web of Conferences* **125**(01008). 10.1051/e3sconf/201912501008 (2019).

[35] Azmeri, Legowo S, Rezkyna N (2020). Interphase modeling of soil erosion hazard using a geographic information system and the universal soil loss equation. J. Chin. Soil Water Conserv..

[36] Gusma F, Azmeri A, Jemi FZ, Rahmatan H (2023). Soil erosion rate and hazard level at the Sianjo-anjo Reservoir watershed in Indonesia. J. Water Land Dev..

[37] Phinzi K, Ngetar NS (2019). The assessment of water-borne erosion at catchment level using GIS-based RUSLE and remote sensing: A review. Int. Soil Water Conserv. Res..

[38] Fenjiro I, Zouagui A, Manaouch M (2020). Assessment of soil erosion by RUSLE model using remote sensing and GIS—A case study of Ziz Upper Basin Southeast Morocco. Forum Geogr..

[39] Berteni F, Dada A, Grossi G (2021). Application of the MUSLE model and potential effects of climate change in a small alpine catchment in northern Italy. Water (Switzerland).

[40] Tan ML, Gassman PW, Srinivasan R, Arnold JG, Yang XY (2019). A review of SWAT studies in Southeast Asia: Applications, challenges and future directions. Water (Switzerland).

[41] Kolli MK, Opp C, Groll M (2021). Estimation of soil erosion and sediment yield concentration across the Kolleru Lake catchment using GIS. Environ. Earth. Sci..

[42] Saptari AY, Supriadi A, Wikantika A, Darmawan S (2015). Remote sensing analysis in RUSLE erosion estimation. Indones. J. Geospat..

[43] Ganasri BP, Ramesh H (2016). Assessment of soil erosion by RUSLE using remote sensing and GIS—A case study of Nethravathi Basin. Geosci. Front..

[44] Ali MG (2021). Estimation of potential soil erosion and sediment yield: A case study of the transboundary Chenab River Cathment. Water (Switzerland).

[45] Patil, M., Patel, R. & Saha, A. Sediment yield and soil loss estimation using GIS based soil erosion model: a case study in the MAN catchment, Madhya Pradesh, India. In sEnvironmental Sciences Proceeding, MDPI, pp. 1–13. 10.3390/ecas2021-10348 (2021).

[46] ESRI Inc. ArcGIS Desktop: Version 10.3.’ Redlands, 2014. [Online]. Available: https://www.esri.com/en-us/arcgis/products/arcgis-desktop/overview

[47] Setyawan C, Lee CY, Prawitasari M (2019). Investigating spatial contribution of land use types and land slope classes on soil erosion distribution under tropical environment. Nat. Hazards.

[48] Wischmeier, W. H. & Smith, D. D. *Predicting Rainfall Erosion Losses: A Guide to Conservation Planning, Agricultur* (U.S. Department of Agriculture, Washington D.C. 1978)

[49] Ali SA, Hagos H (2016). Estimation of soil erosion using USLE and GIS in Awassa catchment, Rift valley, Central Ethiopia. Geod. Reg..

[50] Xu L, Xu X, Meng X (2013). Risk assessment of soil erosion in different rainfall scenarios by RUSLE model coupled with information diffusion model: A case study of Bohai Rim, China. Catena (Amst).

[51] Sulistyo, B., Gunarti, T., Hartono. & Danoedoro, P. Pemetaan faktor C dari data penginderaan jauh, *Manusia dan Lingkungan*, **18** (2011)

[52] Patakamuri SK, Muthiah K, Sridhar V (2020). Long-term homogeneity, trend, and change-point analysis of rainfall in the arid district of ananthapuramu, Andhra Pradesh State, India. Water (Switzerland).

[53] Asdak C (2010). Hydrology and Management of Watersheds.

[54] Arsyad S (2010). Water and Soil Coservation.

[55] Hou J, Wang H, Fu B, Zhu L, Wang Y, Li Z (2016). Effects of plant diversity on soil erosion for different vegetation patterns. Catena (Amst).

[56] Yao X, Yu J, Jiang H, Sun W, Li Z (2016). Roles of soil erodibility, rainfall erosivity and land use in affecting soil erosion at the basin scale. Agric. Water Manag..

[57] Othman AA (2021). New insight on soil loss estimation in the northwestern region of the Zagros fold and Thrust Belt. ISPRS Int. J. Geoinf..

[58] Terranova O, Antronico L, Coscarelli R, Iaquinta P (2009). Soil erosion risk scenarios in the Mediterranean environment using RUSLE and GIS: An application model for Calabria (southern Italy). Geomorphology.

[59] Azmeri A, Nurbaiti N, Mawaddah N, Yunita H, Jemi FZ, Sundary D (2022). Surface erosion hazard and sediment yield for Keuliling Reservoir in Indonesia. J. Water Land Dev..

[60] Ghani A. H. A., Lihan, T., Rahim, S. A., Musthapha, M. A., Idris, W. M. R. & Rahman, Z. A. Prediction of sedimentation using integration of RS, RUSLE model and GIS in Cameron Highlands, Pahang, Malaysia. In *AIP Conference Proceedings*, **1571**(December 2013), 543–548 (2013). 10.1063/1.4858711

[61] Tjasyono, B. H. K., Gernowo, R., Woro, S. B. H. & Ina, J. The characteristics of rainfall in the Indonesian monsoon. In *International Symposium on Equatorial Monsoon System, September* (Yogyakarta, Indonesia, 2008), pp. 16–18

[62] Sujono J, Jayadi R, Nurrochmad F (2018). Heavy rainfall characteristics at south-west of Mt. Merapi-Yogyakarta and central java province, Indonesia. Int. J. GEOMATE.

[63] Tanyaş H, Kolat Ç, Süzen ML (2015). A new approach to estimate cover-management factor of RUSLE and validation of RUSLE model in the watershed of Kartalkaya Dam. J. Hydrol. (Amst).

[64] Kusumandari A (2014). Soil erodibility of several types of green open space areas in Yogyakarta City, Indonesia. Proced. Environ. Sci..

[65] Meek, B. D., Chesworth, W. & Spaargaren, O. Encyclopedia of soil science. In *Encyclopedia of Earth Sciences Series* 605–606 (Springer, Dordrecht, 2008). 10.1007/978-1-4020-3995-9_479.

[66] Thorp, J. Geomorphology. In *Encyclopedia of Earth Science* 10–11 (Springer Berlin Heidelberg, 1968). 10.1007/3-540-31060-6_7

[67] Siswanto, S. Y., Sule, M. I. S. The Impact of slope steepness and land use type on soil properties in Cirandu Sub-Sub Catchment, Citarum Watershed. In *IOP Conference Series Earth Environmental Science*, **393**(1) (2019). 10.1088/1755-1315/393/1/012059.

[68] Nihila A (2012). Water poverty index mapping and GIS-based approach for identifying potential water harvesting sites. Int. J. Remote Sens. Geosci..

[69] Wagari M, Tamiru H (2021). RUSLE model based annual soil loss quantification for soil erosion protection: A case of Fincha catchment, Ethiopia. Air Soil Water Res..

[70] Panagos P, Borrelli P, Meusburger K, Alewell C, Lugato E, Montanarella L (2015). Estimating the soil erosion cover-management factor at the European scale. Land Use Policy.

[71] Bozali N (2020). Assessment of the soil protection function of forest ecosystems using GIS-based multi-criteria decision analysis: A case study in Adıyaman, Turkey. Glob. Ecol. Conserv..

[72] Large River Basin of Serayu Opak. *Rencana Pola Pengelolaan Sumber Daya Air Wilayah Sungai Progo Opak Serang* (2016)

[73] Rezagama A, Sarminingsih A, Zaman B, Handayani DS (2019). Analysis of land use changes effect on erosion and sedimentation potential in Progo watershed. J. Phys. Conf. Ser..

[74] Rassarandi, F. D., Santosa, P. B., & Harintaka. Pemetaan tingkat bahaya erosi menggunakan metode RUSLE (revised universal soil loss equation) dan SIG di Sub DAS Kali Progo Hulu, in *Simposium Nasional Sains Geoinformasi*, **40**, 143–151 (2018)

[75] Hastuti D, Marsono D (2004). Evaluation of Land Use for Land Conservation Direction in the Serang Kulon Progo Watershed, Yogyakarta.

[76] Farhan Y, Nawaiseh S (2015). Spatial assessment of soil erosion risk using RUSLE and GIS techniques. Environ. Earth Sci..

[77] Taghizadeh-Mehrjardi R, Bawa A, Kumar S, Zeraatpisheh M, Amirian-Chakan A, Akbarzadeh A (2019). Soil erosion spatial prediction using digital soil mapping and RUSLE methods for Big Sioux River watershed. Soil Syst..

[78] Abdo H, Salloum J (2017). Spatial assessment of soil erosion in Alqerdaha basin (Syria). Model. Earth Syst. Environ..

[79] Thomas K, Chen W, Lin BS, Seeboonruang U (2020). Evaluation of the sediment delivery distributed (SEDD) model in the Shihmen reservoir watershed. Sustainability (Switzerland).

[80] Wahyuningrum N, Sudira P, Supriyo H, Sabarnurdin S (2014). Perhitungan nilai nisbah hantaran sedimen dengan menggunakan kurva sedimen dan model erosi tanah. Agritech.

[81] Nindito DA, Istiarto I, Kironoto BA (2008). Simulasi numeris tiga dimensi kantong lumpur Bendung Sapon. J. Civ. Eng. Forum.

[82] Wongtragoon, U., Kubo, N. & Tanji, H. A study on improving sand trap in a large scale of. In *2nd World Irrigation Forum*, 1–13 (2016)

[83] Namu PN, Raude JM, Mutua BM (2017). Effects of continuous flushing on the sediment removal efficiency in settling basins of small scale irrigation projects; A case study of Kiriku-Kiende irrigation project, Embu County, Kenya. Int. J. Hydrol..

